# Proteinuria as a Critical Indicator of Kidney Dysfunction in Type 2 Diabetes Patients: Insights From a Cross-Sectional Study

**DOI:** 10.7759/cureus.69946

**Published:** 2024-09-22

**Authors:** Ritah Kiconco, Joash Okoboi, Samuel Mwesige, Kizito Muwonge, Robert Kinobe, Robert Kalyesubula, Gertrude N Kiwanuka

**Affiliations:** 1 Department of Biochemistry, Soroti University, Soroti, UGA; 2 Department of Medical Laboratory Science, Mbarara University of Science and Technology, Mbarara, UGA; 3 School of Medicine, Makerere University College of Health Sciences, Kampala, UGA; 4 Department of Biochemistry, Mbarara University of Science and Technology, Mbarara, UGA

**Keywords:** biomarkers, hyperuricemia, proteinuria, screening tests, urinalysis

## Abstract

Background

Kidney dysfunction is a common finding among patients with diabetes mellitus. We sought to determine the prevalence and contributors to kidney dysfunction among type 2 diabetes mellitus (T2D) patients.

Methods

In this descriptive and analytical cross-sectional study, we received consent and enrolled 148 T2D patients attending the diabetic clinic of Soroti Regional Referral Hospital in eastern Uganda from May 2023 to July 2023. We used questionnaires to obtain participants’ socio-demographic and behavioral characteristics. Blood and urine samples were collected and analyzed for kidney dysfunction and other metabolic biomarkers, which included serum glucose, creatinine, estimated glomerular filtration rate (eGFR), blood urea nitrogen (BUN), uric acid, triglycerides, total cholesterol, low and high-density lipoprotein cholesterol, and urinalysis. We performed blood tests using an automated chemistry analyzer.

Results

Of the 148 participants with T2D, 71 (47.97%) had kidney dysfunction (95% CI: 39.98-56.07). Bivariate and multivariate logistic regression analysis revealed only total proteinuria (OR 1.046; 95% CI: 1.014-1.079; P = 0.004) as a significant factor contributing to the observed kidney dysfunction in T2D patients.

Conclusion

Kidney dysfunction is a common occurrence among T2D patients in our study setting. The presence of proteins in urine contributes immensely to the observed kidney status in our study population. Total proteinuria should be frequently screened in the routine care of T2D patients in eastern Uganda to curb kidney dysfunction at an early stage. Also, a larger study is recommended to validate these findings and inform screening programs in the study area.

## Introduction

Globally, the total number of people with diabetes mellitus (DM) is estimated to increase from 415 million (8.8%) in 2015 to 642 million (10.4%) in 2040, with the largest alterations expected to occur in the urban population of low- to middle-income countries (LMICs) [1,2]. By 2040, the difference in DM prevalence worldwide is projected to broaden, with 477.9 million affected people living in urban areas and 163.9 million in rural areas [2]. 

Kidney dysfunction in diabetic patients is the most common cause of end-stage kidney disease (ESKD) and is associated with increased morbidity and mortality among cases [3]. The prevalence and incidence of ESKD remain high, driven largely by the high prevalence of diabetes and better survival among those with diabetes [4]. It affects more than 10% of the general population worldwide, while people with DM and hypertension are exposed to a 50% risk of developing kidney disease [5]. Between 2002 and 2015, the incidence of diabetes-associated ESKD saw steep increases of approximately 40%-700% in some developed countries. In the same period, the overall incidence of ESKD increased by 11%. However, diabetes-associated ESKD incidence declined and was reported over the same period in other developed countries to be as low as 1% [6]. Previous studies have also demonstrated that the prevalence of type 2 DM-associated kidney dysfunction (T2D-KD) varies substantially among geographical regions [5]. 

It has been postulated that the burden of DM and its complications in LMICs may be attributed to economic development and rapid urbanization as a result of increased caloric intake and the adoption of a sedentary lifestyle [7-9]. More importantly, the most striking demographic change in DM prevalence in global terms seems to be related to the growth of the proportion of the elderly population [10] as well as the increase in the number of chronic and acute diseases in the general population, with profound effects on quality of life, demand for health services, and economic costs [11]. Regardless of intensive treatment with hyperglycemic control, blood pressure control, and the use of renin-angiotensin system blockade, the prevalence of T2D-KD remains high [12].

The pathogenesis of T2D-KD is complex and multifactorial, with genetic and environmental factors involved [13]. These factors have been further categorized as modifiable and non-modifiable. Some of the non-modifiable factors that contribute to T2D-KD include genetics, sex, age, age at onset, and duration of diabetes. On the other hand, some of the modifiable factors include glycemic control, blood pressure, dyslipidemia, a lack of physical activity, and smoking [14]. Other discovered modifiable factors include chronic low-grade inflammation and advanced glycation end products. Proper management of these modifiable risk factors may improve the prognosis of T2D patients at risk of T2D-KD [14]. 

Kidney disease has been associated with most of the all-cause mortality in patients with DM. This has been substantially attributed to the very low level of kidney disease awareness around the world [4]. Hypercholesterolemia and cardiovascular diseases have been noted to escalate the hazard of developing kidney disease, especially among those with T2D [15]. Hyperglycemia is a well-known risk factor for T2D-KD, together with other risk factors like male sex, obesity, hypertension, chronic inflammation, resistance to insulin, hypovitaminosis D, dyslipidemia and some genetic loci and polymorphisms in specific genes [16]. It is believed that the management of modifiable risk factors could help in reducing the incidence of kidney disease among T2D patients. It has been noted that the increasing incidence of obesity and an aging population together could lead to a greater number of people with T2D-KD [17]. Aging and obesity influence approaches to the management of T2D and the accurate assessment of kidney disease [18]. In the current study setting total proteinuria is typically investigated when a diabetic patient complains of symptoms consistent with a urinary tract infection thus neglecting the test’s importance in the routine screening of diabetic patients for possible kidney dysfunction.

In this cross-sectional study, we determined the prevalence of kidney dysfunction and its’ contributors among type 2 diabetic patients at a Soroti regional referral hospital in eastern Uganda. The descriptive aspect characterized the demographic and clinical characteristics of the diabetic patient population, while the analytical component examined the relationships between factors such as total proteinuria and other relevant variables.

## Materials and methods

Study setting and participants

The study was a cross-sectional study. It was conducted at the diabetic clinic of Soroti Regional Referral Hospital (SRRH), 1°42'58.0"N, 33°36'47.0" E (latitude:1.716111; longitude:33.613056) in Uganda. The hospital is located in Soroti City, approximately 321 km from Kampala, Uganda. The facility has a bed capacity of 250 and serves a population of about three million people from 10 districts of the Teso sub-region. The major economic activity of the population is mixed subsistence farming. The population is mainly young (UBOS 2015 census report). A consecutive sampling method was used to recruit study participants until the required sample size was obtained. Participants were 1) all those patients with a T2D diagnosis during the one-month data collection period and 2) given written informed consent to participate. The study excluded 1) pregnant women due to the confounding effect of gestational diabetes (eligible female participants were asked to mention their last normal menstruation period to determine their pregnancy status) and 2) those with known debilitating diseases that would otherwise impede their participation in the current study, such as mental health illnesses. The sample size was calculated using the formula of N=Z^2^pq/e^2^ [19], where z is the z-score, p is the sample proportion, q is the percentage of failure, and e is the margin of error, with an estimated prevalence of T2D-KD which is 10.8% [15]; 5% type I error and 95% confidence interval. The calculated sample size was 148 T2D patients.

General study procedure

We enrolled participants who met the inclusion criteria into the study. We interviewed participants using a pretested questionnaire to obtain their age, sex, year of diabetes diagnosis (recorded as the duration of DM), and lifestyle risk factors, such as smoking and alcohol consumption. We measured blood pressure on the day of enrolment into the study using a mercury sphygmomanometer with small (<21 cm) and normal (22-32 cm) cuff sizes with the left arm at the level of the heart while the patient was seated. Hypertension was defined as systolic blood pressure of ≥ 140 and/or diastolic blood pressure of ≥90mmHg. Participants’ weight was measured using a weighing scale machine, making sure that the patient had no heavy clothing or shoes. Height was measured to the nearest 0.1cm on a vertical wall. The participant’s weight in kilograms and the square of their height in meters were used to calculate their body mass index (BMI). T2D-KD was determined using eGFR of >60mL/min/1.73m^2^ using the updated CKD-EPI formula [20] and urinary albumin creatinine ratio (UACR) of ≥30mg/g calculated from direct measurements of participants’ urine samples for albumin and creatinine using reagent materials from Biozek Medical, Inzek international Trading B.V., Netherlands. The definition of kidney dysfunction is as indicated in a study conducted by Low et al. [21]. Other renal and metabolic markers assessed in blood were BUN, total cholesterol, triglycerides, high-density and low-density lipoproteins, and glucose. Urinalysis was also done semi-quantitatively to determine leukocytes, urobilinogen, microalbumin, total protein, bilirubin, glucose, ascorbic acid, specific gravity, ketones, nitrite, creatinine, pH, blood, and calcium.

Sample handling

A random spot urine sample was collected in a clean, dry screw cap urine container for each participant. Participants were given clear verbal and written instructions on how to collect the required midstream urine sample into the container. Using a vacutainer needle and holder, 4mL of venipuncture blood was drawn into a red-top vacutainer tube. Blood specimens were centrifuged at 12,000 revolutions per minute for five minutes to obtain serum at the SRRH laboratory. Serum samples were transported to Grace Laboratories, a Uganda Allied Health Professionals’ Council-certified laboratory located in Mbale, Uganda, for analysis. Serum or urine was aliquoted into cryovial tubes and stored in freezers at -20°C until analysis in case of deferred analysis. Each participant's vial and cryotube was thawed once at 25°C and then processed. System-compatible reagents were used for all designated analytes in blood and urine. The procedures were quality-controlled using appropriate calibrators and controls. The laboratory standardized all equipment before use and based result interpretation on the reference ranges routinely used in patient care.

Statistical analyses

The prevalence of kidney dysfunction, together with its 95% confidence interval, was obtained as a proportion that was expressed as a percentage. The distribution of the prevalence of kidney dysfunction among T2D patients was compared among variables using a Chi-square test or Fisher’s exact test for categorical variables with frequencies less than 5 in any of the cells after cross-tabulation to determine any statistical difference in the distribution of kidney dysfunction. Bivariate and multivariate logistic regression analyses were done to ascertain the independent variables' relationship with kidney dysfunction. The analyses were performed considering adjustments in the regression models. A p-value of less than 0.05 was considered statistically significant. STATA version 17 (StataCorp LLC, College Station, TX) was used as the data analytical software.

## Results

The distribution of kidney dysfunction in different variables among T2D patients is indicated in Table 1.

**Table 1:** Participants' characteristics and kidney dysfunction in T2D T2D: type 2 diabetes mellitus; DM: diabetes mellitus; SD: standard deviation

Variable	Kidney dysfunction with T2D		
	Normal, n (%)	Abnormal, n (%)	P-value
	N=77 (52.03)	N=71 (47.97)	
Sex			0.590
Male	26 (49)	27 (51)	
Female	51 (54)	44 (46)	
Age (years) Mean (SD)	55.81 (13.9)	52.8 (15.8)	0.230
Residence			0.300
Rural	56 (54.4)	47 (45.6)	
Urban	6 (40.0)	9 (60.0)	
Tribe			0.390
Iteso	69 (52.3)	63 (47.7)	
Kuman	5 (38.5)	8 (61.5)	
Education level			0.510
None	40 (58)	29 (42)	
Primary	23 (50)	23 (50)	
Secondary	11 (44)	14 (56)	
Tertiary	3 (38)	5 (63)	
Marital status			0.260
Single	5 (33)	10 (67)	
Married	52 (58)	38 (42)	
Separated	2 (40)	3 (60)	
Widowed	17 (47)	19 (53)	
Religion			0.230
Catholic	29 (54)	25 (46)	
Protestant	25 (44)	32 (56)	
Moslem	2 (40)	3 (60)	
Pentecostal	21 (66)	11 (34)	
Family type			0.049
Extended	6 (60)	4 (40)	
Nuclear	57 (58)	42 (42)	
Single parent	11 (33)	22 (67)	
Current blood pressure			0.420
Normal	34 (49)	35 (51)	
Abnormal	42 (56)	33 (44)	
Physical exercise			0.820
No	59 (52.2)	54 (47.8)	
Yes	17 (50.0)	17 (50.0)	
Hypertension			0.088
No	55 (57)	41 (43)	
Yes	22 (42)	30 (58)	
Diabetic retinopathy			0.720
Yes	13 (50.0)	13 (50.0)	
No	63 (53.8)	54 (46.2)	
Diabetic therapy			0.170
Insulin therapy	17 (42.5)	23 (57.5)	
Oral hypoglycemic	58 (55.2)	47 (44.8)	

Out of the 148 participants, 71 had kidney dysfunction, giving an overall prevalence of 47.97% with a 95% CI of 39.98% to 56.07%. In Table 2, after regression analysis, urinary total proteins had a significant association with kidney dysfunction among the study participants.

**Table 2:** Bivariate and multivariate analysis of contributors to kidney dysfunction among T2D patients CI: confidence interval, DM: diabetes mellitus, eGFR: estimated glomerular filtration rate, HDL: high-density lipoproteins, LDL: low-density lipoproteins; T2D: type 2 diabetes mellitus

Variable	Bivariate analysis		Multivariate analysis	
	Odds ratio (95%CI)	P-value	Odds ratio (95%CI)	P-value
Sex				
Male	1.00		1.00	
Female	0.83 (0.42 – 1.63)	0.589	0.89 (0.29 – 2.62)	0.827
Age	0.99 (0.97 – 1.00)	0.225	0.99 (0.94 – 1.02)	0.278
Education level				
None	1.00			
Primary	1.38 (0.65 – 2.92)	0.401		
Secondary	1.76 (0.70 – 4.42)	0.232		
Tertiary	2.30 (0.51 – 10.40)	0.280		
Marital status				
Single	1.00			
Married	0.37 (0.11 – 1.16)	0.087		
Separated	0.75 (0.09 – 6.04)	0.787		
Widowed	0.56 (0.16 – 1.96)	0.364		
DM duration	0.97 (0.90 – 1.05)	0.445	1.00 (0.89 – 1.13)	0.989
Ever smoked				
No	1.00			
Yes	1.09 (0.21 – 5.58)	0.919		
Herbs use with DM				
No	1.00		1.00	
Yes	0.65 (0.29 – 1.42)	0.278	0.53 (0.18 – 1.57)	0.251
Known hypertensive				
No	1.00		1.00	
Yes	1.92 (0.96 – 3.82)	0.064	1.76 (0.53 – 5.88)	0.356
Current blood pressure				
Normal	1.00		1.00	
Abnormal	0.76 (0.40 – 1.47)	0.420	0.60 (0.200 - 1.82)	0.371
Serum urea	1.01 (0.83 – 1.23)	0.900	1.04 (0.74 – 1.44)	0.832
Serum uric acid	0.98 (0.84 – 1.14)	0.808	0.80 (0.60 – 1.05)	0.112
Total cholesterol	0.75 (0.54 – 1.03)	0.078	0.43 (0.13 – 1.42)	0.166
Triglycerides	1.29 (0.89 – 1.88)	0.181	1.95 (0.96 – 3.99)	0.066
HDL – cholesterol	0.75 (0.35 – 1.61)	0.461	1.62 (0.33 –7.82)	0.551
LDL – cholesterol	0.79 (0.50 – 1.25)	0.305	2.80 (0.48 –16. 21)	0.250
Serum glucose	0.99 (0.93 – 1.47)	0.641	1.06 (0.92 – 1.23)	0.422
Waist circumference	0.99 (0.72 – 1.01)	0.606	0.99 (0.95 – 1.03)	0.607
Waist height ratio	0.47 (0.02 – 14.70)	0.670		
Body mass index	1.03 (0.95 – 1.13)	0.451	1.07 (0.915 – 1.26)	0.380
Dyslipidemia				
Absent	1.00			
Present	1.15 (0.57 – 2.14)	0.690		
Diabetic retinopathy				
No	1.00			
Yes	1.27 (0.54 – 2.98)	0.581		
Anti-DM therapy				
Oral hypoglycemic	1.00			
Insulin therapy	1.54 (0.75 – 3.19)	0.241		
Urinalysis				
Total proteins	1.05 (1.03 – 1.08)	<0.001	1.04 (1.01 – 1.08)	0.004
Glucose	0.99 (0.99 – 1.00)	0.529	1.00 (0.994 –1.002)	0.915
Ascorbic acid	1.02 (0.99 – 1.04)	0.236	0.99 (0.95 – 1.02)	0.538
Ketones	0.98 (0.95 – 1.02)	0.382	0.94 (0.81 – 1.10)	0.455
pH	1.20 (0.96 – 1.50)	0.110	1.24 (0.91- 1.68)	0.170
Blood	1.03 (0.99 – 1.07)	0.112	1.02 (0.98 – 1.07)	0.298
Calcium	1.02 (0.93 – 1.12)	0.668	1.04 (0.89 – 1.21)	0.648

## Discussion

Our study has revealed a high prevalence of kidney dysfunction among T2D patients receiving healthcare at Soroti Regional Referral Hospital’s diabetic clinic, at 47.97% out of the 148 study participants who were tested for kidney dysfunction. Several studies in the recent past have also documented the prevalence of kidney dysfunction by screening for micro- and macroproteinuria. The frequency of microalbuminuria and macroalbuminuria among patients with T2D was found to be 15.2% (54/355) in a Japanese study that examined the prevalence of albuminuria, renal dysfunction, and associated clinical factors in Japanese patients [19]. Differences in sample sizes, the way that micro- and macroalbuminuria are defined, and racial differences may all contribute to the disparity in prevalence statistics. Another cross-sectional study looking at the prevalence of kidney dysfunction and associated factors in Chinese individuals with T2D found the prevalence of albuminuria to be 27.1% out of the 3,310 patients who were sampled [20].

Like in the previous study, the difference in albuminuria could be due to the difference in the definition of albuminuria, the sample size tested, and race. In a closely related study, the prevalence of microalbuminuria, which is used to infer kidney dysfunction in diabetic patients, was found to be 22.1% out of 140 patients who were sampled at Mbarara Regional Referral Hospital. The study examined microalbuminuria and the traditional serum markers of diabetic nephropathy [21]. This prevalence statistic is smaller than what we have in the current study, probably due to the difference in the methods that were used to quantify albuminuria or the differences in the ethnicities of the two populations. Two separate studies were conducted to determine the incidence, time to occurrence, and predictors of chronic kidney disease (CKD) in type 2 diabetic patients in Ethiopia. The researchers found that one out of every 10 diabetic patients experienced CKD, and the median time to develop CKD was five years [15,17]. Another cross-sectional study on patients (n = 1,861) aged 21 to 89 years with T2D who had attended the DM center of a single acute care public hospital or a primary care polyclinic in the northern region of Singapore found the prevalence of kidney disease in patients with T2D to be high, that is, 53% [22]. According to reports, the prevalence of kidney disease among people with T2D, defined as having an ACR of ≥30 mg/g or a GFR of <60 mL/min per 1.73 m^2^, varies between 27.9% and 77% across numerous nations. This wide variation in the burden of kidney disease in these studies could be due to differences in definitions of kidney disease or patient profiles such as ethnicity and healthcare setting [22]. The prevalence of kidney dysfunction among the study participants did not significantly differ when categorized by age or sex.

In the logistic regression analysis shown in Table 2, total proteinuria was considered a contributor to the presence of kidney dysfunction among the T2D patients in this study population. This finding agrees with several other studies that have been done to identify the role of protein excretion in the pathogenesis of kidney function among diabetic patients. A review study noted that albumin, a pro-inflammatory and pro-fibrotic protein, present in the glomerular filtrate indicates kidney damage [23]. Moreover, it has been noted that a reduction in total proteinuria is associated with fewer adverse renal outcomes [24]. This therefore implies that regular assessment of proteins in urine should be upheld in routine care for T2D patients for early identification of kidney dysfunction among these patients.

In the current study, factors like BMI, serum glucose, urea, and uric acid levels as well as anthropometric measurements like waist circumference and waist-hip ratio, did not have a significant relationship with the presence of kidney dysfunction among T2D patients. This finding does not negate the fact that these factors do not have an active role in the processes leading to kidney dysfunction among the study population. Moreover, there is a study that has implicated obesity and its indicators, such as BMI, waist-hip ratio, and waist-height ratio, to contribute to the presence of albuminuria, a marker for kidney dysfunction, among T2D patients [25]. A cross-sectional study on patients (n = 1,861) aged 21 to 89 years with T2D who had attended the diabetic clinic of a single acute care public hospital or a primary care polyclinic in the northern region of Singapore had the following as significant risk factors for kidney disease: neuropathy; hypertension, hypertriglyceridemia, obesity; duration of T2D, poor glycemic control, ageism/advanced age, cardiovascular disease and proliferative retinopathy [22,26].

Although we found no association between aging and kidney dysfunction among type 2 diabetic patients, some studies implicated it. A study evaluating the prevalence of CKD and its progression rate in elderly patients with T2D found the condition was a common occurrence in older patients, and the progression rate to CKD is also high, especially in those with diabetes duration ≥10 years [27]. Contrary to what other studies have found, a cross-sectional study aimed to investigate the prevalence and risk factors of chronic kidney disease among 1,096 primary care T2D patients in northern Thailand did not find an association between hypertension or blood pressure and the risk of CKD among T2D patients [10].

As a limitation of this study, we used one spot urine sample and not 24-hour urine samples to deduce microalbuminuria. However, it has been studied that one-spot urine samples assessing the albumin-creatinine ratio can equally yield accurate and acceptable results [23]. Glycated hemoglobin (HbA1c) as a marker of glycemic control was not one of the variables we assessed in this study, yet we acknowledge that glycemic control among T2D patients could have an association with kidney dysfunction among this population. If assessed, HbA1c results could be compared between the controlled and uncontrolled T2D patients. Due to the cross-sectional nature of the study, the definition of hypertension was based on a one-time measurement of a participant’s blood pressure which is unreliable for a conclusive hypertension diagnosis. Also, due to recall bias and missing records in the patient files, it was difficult to ascertain whether a participant was on any hypertensive treatment and for how long.

## Conclusions

Kidney dysfunction is a common complication among individuals with T2D. Our study found that the presence of protein in the urine (total proteinuria) is a major contributor to the observed kidney dysfunction in this population. To address this issue, we recommend that routine screening for total proteinuria should be implemented in the care of T2D patients in eastern Uganda. Early detection and management of this biomarker could help prevent or delay the progression of kidney disease in this high-risk group. Additionally, a large-scale study is warranted to further validate these findings and inform the development of targeted screening and intervention programs in the study region.
